# The impact of carvacrol on the larval gut bacterial structure and function of *Lymantria dispar*

**DOI:** 10.3389/fmicb.2024.1417598

**Published:** 2024-09-18

**Authors:** Jing Yang, Yun-Ze Chen, Guo-Cai Zhang

**Affiliations:** ^1^College of Forestry, Guizhou University, Guiyang, China; ^2^College of Biological Sciences, Guizhou Education University, Guiyang, China; ^3^College of Forestry, Northeast Forestry University, Harbin, China

**Keywords:** carvacrol, *Lymantria dispar*, gut bacterial community, 16S rRNA, mode of action

## Abstract

**Introduction:**

The gut bacteria of insects play an important role in regulating their metabolism, immune system and metabolizing pesticides. Our previous results indicate that carvacrol has certain gastric toxic activity on *Lymantria dispar* larvae and affects their detoxification metabolism at the mRNA level. However, the impact of carvacrol on the gut bacteria of *L. dispar* larvae has been unclear.

**Methods:**

In this study, the 16S rRNA sequencing technology was used to sequence and analyze the gut bacteria of the larvae which were exposed with sublethal concentration (0.297 mg/mL) and median lethal concentration (1.120 mg/mL), respectively.

**Results:**

A total of 10 phyla, 16 classes, 47 orders, 72 families, 103 genera, and 135 species were obtained by using a 97% similarity cutoff level. The dominant bacterial phyla in the gut of the *L. dispar* larvae are *Firmicutes* and *Proteobacteria*. The treatment with carvacrol can significantly affect the structure of gut bacteria in the larvae of the *L. dispar*. At both doses, carvacrol can shift the dominant gut bacteria of the larvae from *Proteobacteria* to *Firmicutes*. At the genus level, two doses of carvacrol can significantly enhance the relative abundance of probiotic *Lactobacillus* in the gut of *L. dispar* larvae (*p* ≤ 0.01). Additionally, significant differences were observed among the five bacterial genera *Burkholderia-Caballeronia-Paraburkholderia*, *Anoxybacillus*, *Pelomonas*, *Mesorhizobium* (*p* ≤ 0.05). The analysis of *α*-diversity and *β*-diversity indicates that the treatment with carvacrol at two doses significantly affect the bacterial richness and diversity in the larvae. However, the results of functional classification prediction (PICRUSt) indicate that carvacrol significantly down-regulate 7 functions, including Energy metabolism, Cell growth and death, and up-regulate 2 functions, including Carbohydrate metabolism and Membrane transport. The network analysis indicates that the correlation between gut bacteria also has been changed. In addition, the insecticidal activity results of carvacrol against *L. dispar* larvae with gut bacteria elimination showed that gut bacteria can reduce the insecticidal activity of carvacrol against *L. dispar* larvae.

**Discussion:**

This study provides a theoretical foundation for understanding the role of gut bacteria in detoxifying plant toxins and conferring pesticide resistance.

## 1 Introduction

As a worldwide forestry leaf-eating pest, *Lymantria dispar* Linnaeus (Lepidoptera: Liparidae) are mainly distributed in temperate regions of the Northern Hemisphere, such as China, South Korea, North America, and Europe (Song et al., 2022; Xu et al., 2023). The larvae of *L. dispar* harms up to 2 million acres annually in eastern and northern China (Sun et al., 2014). The larvae of *L. dispar* can harm more than 600 host plants including poplar, oak, and birch (Cao et al., 2015; Srivastava et al., 2020). When trees are not enough to provide sufficient food sources, the larvae of *L. dispar* can also harm crops and rank grasses. At present, the most effective method for controlling the *L. dispar* is through the use of chemical pesticides, however the harm caused by the irrational use of chemical pesticides has attracted more and more attention (Zhang et al., 2023). Just like using organochlorine, organophosphorus, and pyrethroids to control the *Anaphothrips obscurus* in the order Thysanoptera, the resistance and environmental adaptability of pests to insecticides rapidly increase after control (Broadbent and Pree, 1997; Zhao et al., 1995). Therefore, exploring the effect of bioderived compounds on the control of *L. dispar* can provide a new way to alleviate the phenomenon of drug resistance in *L. dispar.*

As a monoterpene compound, carvacrol (2-methyl-5-isopropylphenol) is widely present in the essential oils of Lamiaceae family plants such as *Origanum vulgar*, *Thymus mongolicus*, and *Satureja hortensis* (Moazeni et al., 2014; Youssefi et al., 2019). Carvacrol is mainly used to prepare essences such as dill, clove, mint, and vanilla, as well as toothpaste, tooth powder, oral products, talcum powder, soap, and daily industrial products. Previous studies indicated that carvacrol may have potential toxicity risks, such as causing oxidative damage to cells (Fuentes et al., 2021; Llana-Ruiz-Cabello et al., 2014). However, it is generally safe (Konig et al., 2023) while the only concern is that it is corrosive. In addition, carvacrol can kill bacteria and intestinal parasites, so it can be also used as a disinfectant and fungicide. Studies have shown that carvacrol has certain effects on a variety of agricultural and forestry pests including, Coleopteran, Homopteran, Dipteran, Lepidopteran, Neuropteran, and Hemipteran (Konecka et al., 2020). Carvacrol show fumigant activity against *Tribolium castaneum*, and when the dose in the air is 46.2 mg/L, it has a significant inhibitory effect on the hatching of *T. castaneum* eggs (Erler, 2005). Studies have shown that the essential oil of *S. montana* and its main component carvacrol have good stomach toxicity against the *Acrobasis advenella* and can inhibit the growth of the larvae. In addition, carvacrol can significantly activate the enzyme activities of catalase, *α*-glucosidase, and *β*-glucosidase in the larvae (Magierowicz et al., 2019). Studies have shown that the synergistic combination of carvacrol and *Bacillus thuringiensis* crystalline proteins (Cry) has a cooperative effect, capable of reducing the damage to crops caused by *Cydia pomonella* and *Spodoptera exigua* (Konecka et al., 2020). The nicotinic acetylcholine receptor (nAChRs) may be the target of the insecticidal activity of carvacrol (Tong et al., 2013). As an important symbiotic partner in insects, microorganisms in the insect are often believed to reduce the damage caused by toxins to the host (Siddiqui et al., 2022).

A large number of microorganisms in the gut of insects, including bacteria, fungi, protozoa, viruses, and archaea, among which bacteria dominate the total gut microbiota, accounting for over 99% (Qin et al., 2010). In the process of co evolution with host insects, gut bacterial have formed diverse bacterial community structures and biological characteristics, which have important impacts on the survival, reproduction, growth and development, immune protection, and detoxification metabolism of insects (Wang et al., 2020). The composition of insect gut bacteria is not fixed and can be influenced by factors such as diet, environment, and developmental stage (Bai et al., 2023). The gut bacterial can aid their host insects in reducing the harm from toxic plant secondary metabolites and pesticides (Briones-Robiero et al., 2017). As a plant secondary metabolite widely present in Salix and Populus plants, salicin can reduce the weight gain and survival rate of *Tenebrio molitor* larvae treated with antibiotics to eliminate gut bacteria, indicating that gut bacteria play an important role in degrading heterologous toxic substances (Genta et al., 2006). Studies has shown that treating *Spodoptera frugiperda* (Lepidoptera: Noctuidae) larvae with two sublethal doses of emamectin benzoate and tetrachlorantraniliprole for 24 h, the composition and diversity of gut bacteria in the *Spodoptera frugiperda* larvae has been changed. Compared with the control group, *S. frugiperda* larvae was able to significantly upregulate the relative abundance of *Burkholderia-Caballeronia-Paraburkholderia*, *Stenotrophobacter*, *Nitrospira*, *Blastocatella*, *Sulfurifusis*, and *Flavobacterum*, in order to reduce degradation or detoxification of emamectin benzoate and tetrachlorantraniliprole (Chang et al., 2023). The current existing research focuses on the gut bacterial of the *L. dispar.* Adversity stressors (acute temperature, feed pH, and manganese ions) can disrupt the structure and function of the gut bacterial communities in the gut of the *L. dispar* (Zeng et al., 2021; Zeng et al., 2020a; Zeng et al., 2019). Infection with *Beauveria bassiana* spores may disrupt the bacterial ecology of the gut in *L. dispar* larvae, which could be attributed to a reduction in the intestinal content of hydrogen peroxide (Bai et al., 2020). Two sub lethal doses of plant secondary metabolites, nicotine and aconitine, were used to treat the 4th instar larvae of the *L. dispar* for 72 h. The dominant bacteria and structure of the gut bacteria of the *L. dispar* larvae has been changed. The ability of the gut bacteria to decompose secondary metabolites and the nucleotide transport function were, respectively, affected by nicotine and aconitine, thereby affecting the growth and even death of the larvae (Zeng et al., 2020b).

In our previous study, we found that carvacrol had growth inhibition, a decrease in food intake, and ultimately death on the larvae of *L. dispar*, it also could affect the detoxification of *L. dispar* larvae at the mRNA level (Chen et al., 2022; Chen et al., 2021). However, there are few reports on the impact of carvacrol on the gut bacterial of the *L. dispar*. Therefore, we treated the third instar larvae of *L. dispar* with sublethal and median lethal doses, respectively, and utilized high-throughput sequencing technology to examine the impact of carvacrol on the gut bacteria of the *L. dispar*. The aim of this study was to analyze the effects of carvacrol on the structure, diversity, function and interactions of gut bacteria in the larvae of the *L. dispar*. These findings provided a theoretical foundation for elucidating the physiological and biochemical responses of the *L. dispar* larvae to carvacrol stress and offer a basis for the control of *L. dispar*.

## 2 Materials and methods

### 2.1 Insect rearing and reagents

The eggs masses of *Lymantria dispar* were collected from Harbin experimental forest farm of Northeast Forestry University (Harbin, China) and were placed in an artificial climate incubator with a culture temperature of (25 ± 1)°C, a photoperiod of (14L: 10D), and a relative humidity of (75 ± 1)% for cultivation (Cao et al., 2015). The healthy 3rd instar larvae were randomly selected for subsequent experiments. The artificial feed for *L. dispar* were purchased from the Institute of Forest Ecological Environment and Protection of the Chinese Academy of Forestry (Beijing, China). Carvacrol (CAR) was purchased from Macleans Biochemical Technology Co., Ltd. (Shanghai, China). Dimethyl sulfoxide (DMSO) was purchased from Sinopharm Chemical Reagent Co., Ltd. (Shanghai, China). Tetracycline, ampicillin, chloramphenicol, and Kanamycin sulfate were purchased from Beijing Bio-Top Technology Co., Ltd. (Beijing, China).

### 2.2 Collection of gut bacteria samples

A total of 270 third larvae were randomly divided into 9 groups for treatment with carvacrol at 72 h. Feed mixing method was used to treat *L. dispar* larvae (Zeng et al., 2021). Based on our previous bioassay results, the treatment doses were sublethal dose (LC_20_ = 0.297 mg / mL) and median lethal dose (LC_50_ = 1.120 mg/mL) (Chen et al., 2021). The CAR was administered through a mixed feed method as previously described. The distilled water contained the same volume of 10% (v/v) DMSO was used as a control group. The larvae of *L. dispar* were used for gut bacteria analysis were divided into three groups (LC_20_ treatment group, LC_50_ treatment group, and the control group). Each group contained three replicates, each replicate contained the midgut tissue of five larvae (Wu et al., 2022). After the surface of the surviving *L. dispar* larvae at 72 h were wiped with 75% ethanol and physiological saline, the *L. dispar* larvae were placed in a super clean workbench for dissection. The intestinal solutes of the *L. dispar* were washed with sterile physiological saline, and then the guts of the larvae were frozen in liquid nitrogen for the determination of 16S rRNA gene sequencing.

### 2.3 Elimination of intestinal bacteria and related biological activity assay

Four antibiotics were used in combination to remove intestinal bacteria. Solutions of tetracycline, ampicillin, chloramphenicol, and kanamycin sulfate with a concentration of 40 mg/mL were prepared separately in sterile water. After thoroughly mixing the solutions, 2.2 mL of the mixture was taken and added to the artificial feed according to the feed mixing method, resulting in a final concentration of 4 mg/g of antibiotics in the artificial feed. After hatching, the larvae have been raised on artificial feed containing antibiotics. When the *L. dispar* larvae turn into 3rd-instar, the increase in weight and survival rate of the larvae were observed and recorded. According to the method of Chen et al. (2021), the insecticidal activity of carvacrol against the larvae of the *L. dispar* eliminated by intestinal bacteria was determined.

### 2.4 16S rRNA gene sequencing

The gut bacteria DNA of *L. dispar* larvae were extracted according to the manufacturer’s instructions of the FastDNA tissue extraction kit (MP Biomedicals, United States), the dose and quality of the *L. dispar* DNA were detected by ultra-micro spectrophotometer (NanoDrop2000, Thermo Fisher Scientific, United States). According to the instructions of the FastPfu DNA polymerase kit (FastPfu Polymerase, TransGen, China), the extracted DNA was subjected to 16S rRNA V3-V4 region fragment PCR amplification. The amplification procedure was as follows: 95°C pre-denaturation for 3 min, 27 cycles of (95°C denaturation for 30 s, 55°C annealing for 30 s, and 72°C extension for 30 s), followed by 72°C stable extension for 10 min, and finally, storage at 4°C. The primers used for amplifying 16srRNA (V3-V4 region) were 338F (ACTCCTACGGGGGGCAG) and 406R (GGACTACHVGGGTWTCTAAT). The PCR product were purified according to the instructions of the AxyPrep DNA gel extraction kit (Axygen Biosciences, Axygen, United States), and the purified product were placed in the micro fluorometer for quantitative detection (Quantus Fluorometer, Promega, United States). The Miseq library of amplified products were constructed according to the instructions of the library building kit (NEXTFLEX Rapid DNA-Seq Kit, Bioo Scientific, United States), and sequence the qualified library according to the instructions of the Miseq sequencing kit (MiSeq Reagent Kit v3/NovaSeq Reagent Kits, Illumina, United States). The libraries were sequenced on Miseq PE300 (Illumina Miseq, Illumina, United States).

### 2.5 Statistical analyses

The original sequencing sequence was subjected to quality control by removing bases with a tail mass value of less than 20 and containing N-containing through Fastp^1^ (Chen et al., 2018). Paired reads that meet quality control requirements were spliced by FLASH software^2^ (Magoc and Salzberg, 2011). Sequences with a similarity greater than 97% were subjected to OTU clustering by UPARSE^3^ (Edgar, 2013; Stackebrandt and Goebel, 1994). Each sequence was annotated for species classification by using RDP classifier,^4^ and was comparied with the Silva 16S rRNA database (v138), with a matching threshold set at 70% (Wang et al., 2007). Species with sequence numbers greater than or equal to 5 in all three replicates were retained, and the phylum *Cyanophyta* was deleted due to being considered feed contamination. In addition, the Mitochondrial and Chloroplast gene sequences were removed.

The R language software package (version 3.3.1) was utilized to count the number and species of OTU in each sample. The *α* and *β* diversities of the samples were estimated at the OTU level by used MOTHUR and UniFrac, respectively (Wang et al., 2023). PICRUSt (Phylogenetic Investigation of Communities by Reconstruction of Unobserved States) was utilized for KEGG functional annotation of OTU, thereby predicting the functions of gut bacteria (Langille et al., 2013). The Spearman correlation coefficient was used to calculate the correlation at the genus level and the network topological properties was calculated by Gephi (Jiang et al., 2017). The significant results of bacterial abundance, diversity, and function prediction were calculated by the one-way ANOVA variance analysis method in SPSS 19.0, where *p* < 0.05 indicated significant differences. The R language software package (version 3.3.1) was utilized for statistical analysis and plotting. The above bioinformatics analysis of the structure and function of gut bacteria in *L. dispar* larvae was completed through the Shanghai Meiji Information Cloud Platform^5^ (Ma et al., 2018).

## 3 Results

### 3.1 Sequencing data statistics and clustering

**Figure 1:**
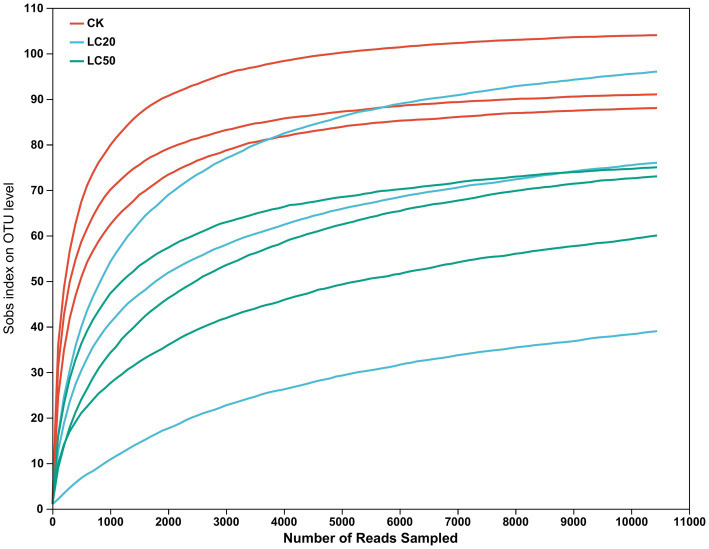
Rarefaction curve of gut bacterial species in the 3rd instar larvae of *L. dispar* (CK: control group; LC20: treatment group with 0.0297 mg/mL of CAR; LC50: treatment group with 1.120 mg/mL of CAR).

After quality filtering and removal of redundant sequences, a total of 453,624 valid sequences were obtained, with an effective base number of 193,180,336 bp and an average effective sequence length of 425.87 bp (Table 1). The rarefaction curve results indicated that as the number of reads sampled increased, the number of OTUs tended to stabilize, and proved this sequencing data is sufficient (Figure 1).

**Table 1:** Sequencing statistics of gut bacteria in *L. dispar* with different group.

Sample name	Sequencing number	Base number	Mean length	Min length	Max length	OTU number
LC_50__1	42,937	18,307,612	426.38	396	431	75
LC_50__2	69,410	29,345,651	422.79	239	431	60
LC_50__3	61,768	26,360,631	426.77	208	430	73
LC_20__1	51,206	21,866,811	427.04	388	431	76
LC_20__2	57,234	24,443,441	427.08	262	431	96
LC_20__3	54,896	23,490,334	427.91	262	431	39
CK_1	38,927	16,481,866	423.40	232	492	91
CK_2	34,866	14,846,775	425.82	200	441	104
CK_3	42,380	18,037,215	425.61	262	430	88
Total	453,624	193,180,336	425.87	200	492	137

CK: control group; LC_20_: treatment group with 0.0297 mg/mL of CAR; LC_50_: treatment group with 1.120 mg/mL of CAR. The numbers 1–3 following the sample names represent different replicates, respectively.

### 3.2 Analysis of differences in gut bacteria composition

**Figure 2:**
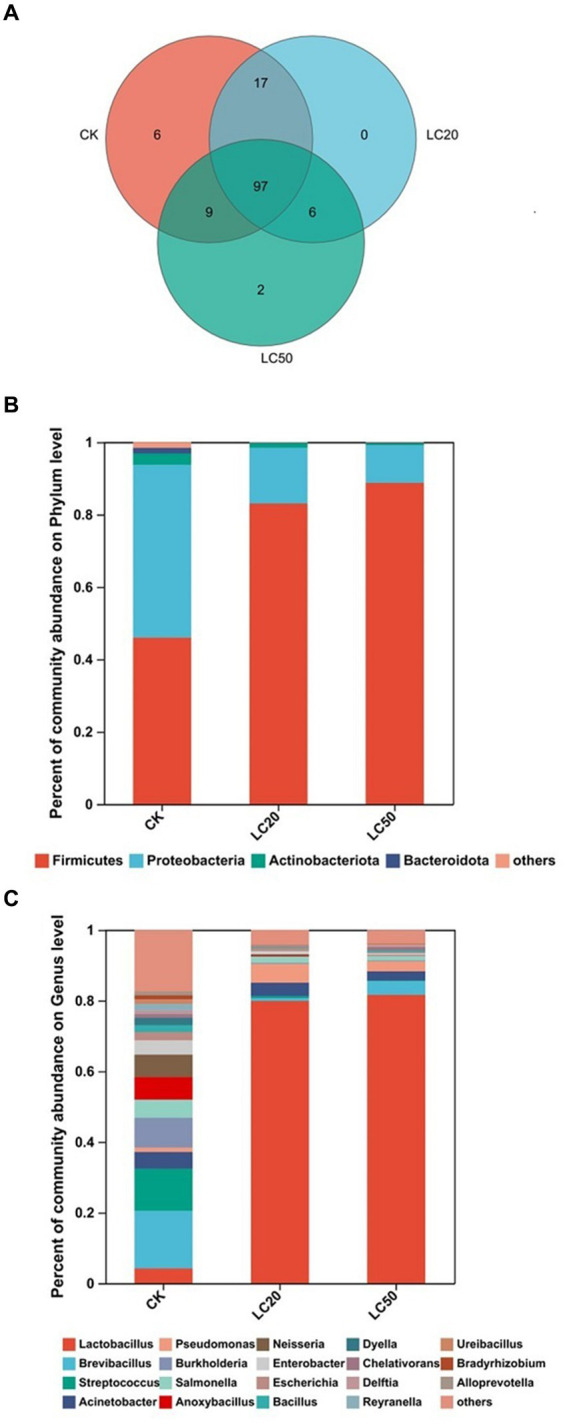
Gut bacterial composition analysis of the control group and the treatment group. **(A)** Venn diagram based on OTU level treatment group and control group; **(B)** Relative abundances of bacterial communities at phylum levels; **(C)** Relative abundances of bacterial communities at genus levels. CK: control group; LC20: treatment group with 0.0297 mg/mL of CAR; LC50: treatment group with 1.120 mg/mL of CAR.

A total of 137 OTUs were obtained by clustering, including 10 phyla, 16 classes, 47 orders, 72 families, 103 genera, and 135 species. The number of common and unique OTUs to three groups was statistically analyzed (Figure 2A). In addition to 97 OTUs shared between the three groups. A total of 17 OTUs were present in LC20 treatment group and the control group. There were 9 OTUs in the LC50 treatment group and the control group.

Based on phylum level, the bacterial composition of the three groups was analyzed (Figure 2B). The dominant bacteria in the control group were Proteobacteria, with a relative abundance of 47.71%. The dominant bacteria in the treatment groups with LC20 and LC50 of CAR were Firmicutes, with relative abundance of 83.06, and 88.74%, respectively. At the genus level (Figure 2C), the dominant bacteria in the control group were *Brevibacillus* (16.31%). The dominant bacteria in LC20 and LC50 treatment group were *Lactobacillus*, with relative abundance of 79.89 and 81.63%, respectively. The results indicated that carvacrol could affect the relative abundance of gut bacteria in the *L. dispar* larvae at the phylum and genus levels.

**Figure 3:**
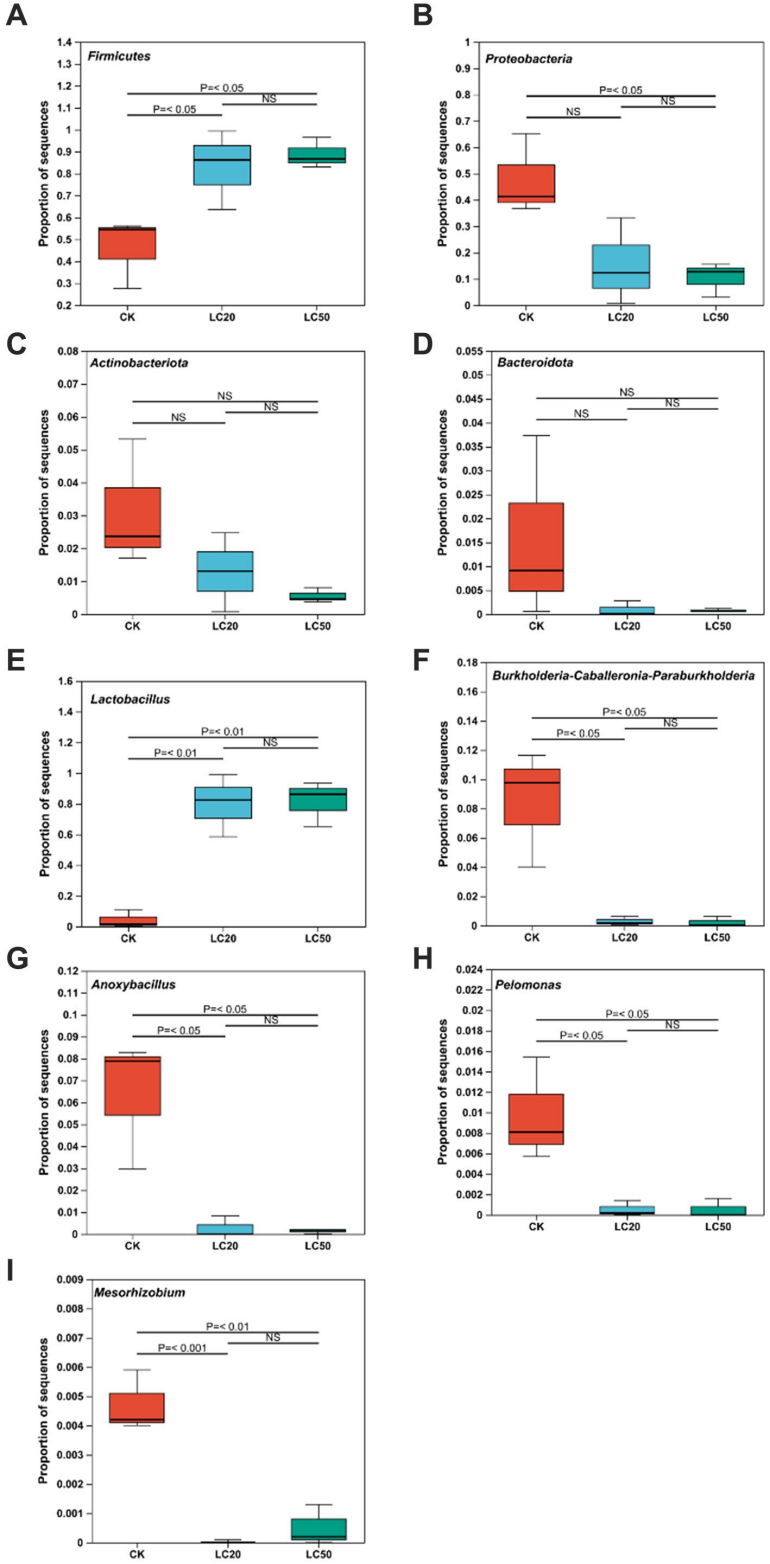
Analysis of the significance of relative abundance difference between groups at phylum and genus levels. *p* ≤ 0.05 indicates significant difference; *p* ≤ 0.01 indicates extremely significant difference, NS indicates no significant difference. **(A)** The comparison results of *Firmicutes*. **(B)** The comparison results of *Proteobacteria.* **(C)** The comparison results of *Actinobacteria*. **(D)** The comparison results of *Bacteroidota*. **(E)** The comparison results of *Lactobacillus.* **(F)** The comparison results of *Burkholderia-Caballeronia-Paraburkholderia*. **(G)** The comparison results of *Anoxybacillus*. **(H)** The comparison results of *Pelomonas*. **(I)** The comparison results of *Mesorhizobium*. CK: control group; LC20: treatment group with 0.0297 mg/mL of CAR; LC50: treatment group with 1.120 mg/mL of CAR.

The one-way ANOVA of variance was used to determine whether CAR had significant differences at phylum and genus levels in the dominant bacteria in the gut bacterial of *L. dispar* larvae (Figure 3). At the phylum level, compared with the control group, the relative abundance of *Firmicutes* in the gut of *L. dispar* larvae was significantly increased in the two doses of CAR treatment groups (*p* ≤ 0.01) (Figure 3A). Compared with the control group, the relative abundance of *Proteobacteria* in the gut of *L. dispar* larvae treated with LC_50_ was significantly decreased (*p* ≤ 0.01), but there was no significant difference between the two doses of CAR treatment groups (*p* > 0.05) (Figure 3B). There was no significant difference at the relative abundance of *Actinobacteriota* and *Bacteroidota* between the two doses of CAR treatment groups and control group (Figures 3C,D). At the genus level, the relative abundance of *Lactobacillus* (*p* ≤ 0.01) could be significantly increased in the two doses of CAR treatment groups, and the relative abundance of *Burkholderia-Caballeronia-Paraburkholderia*, *Anoxybacillus*, and *Pelomonas* could be significantly downgraded (*p* ≤ 0.01). Interestingly, the relative abundance of *Mesorhizobium* in the LC_20_ dose group was extremely significant different from that in the control group (*p* ≤ 0.001), while the LC_50_ dose group was significantly different from that in the control group (*p* < 0.01).

### 3.3 Richness and diversity analysis

The structural differentiation of bacteria under different treatments could be compared by comparing the community richness (Chao and ACE index) and diversity (Shannon and Simpson index) of bacteria. All midgut tissue samples of *L. dispar* larvae had good richness and diversity (Table 2). The one-way ANOVA analysis of variance was used for significant difference comparison, and the FDR inter group difference test method was used for multiple test correction.

**Table 2:** Diversity and richness of gut bacteria communities of *L. dispar* larvae with different group.

Sample name	Shannon	Simpson	ACE	Chao	Coverage
LC_50__1	1.88	0.33	79.75	79.67	0.99
LC_50__2	1.21	0.49	72.69	73.13	0.99
LC_50__3	0.9	0.63	79.21	76.00	0.99
LC_20__1	1.24	0.58	84.19	84.67	0.99
LC_20__2	1.80	0.37	102.36	105.43	0.99
LC_20__3	0.11	0.97	52.67	52.00	0.99
CK_1	2.97	0.15	91.81	91.14	0.99
CK_2	3.52	0.05	105.13	104.37	0.99
CK_3	2.65	0.16	89.18	90.00	0.99

CK: control group; LC20: treatment group with 0.0297 mg/mL of CAR; LC50: treatment group with 1.120 mg/mL of CAR. The numbers 1–3 following the sample names represent different replicates, respectively.

**Figure 4:**
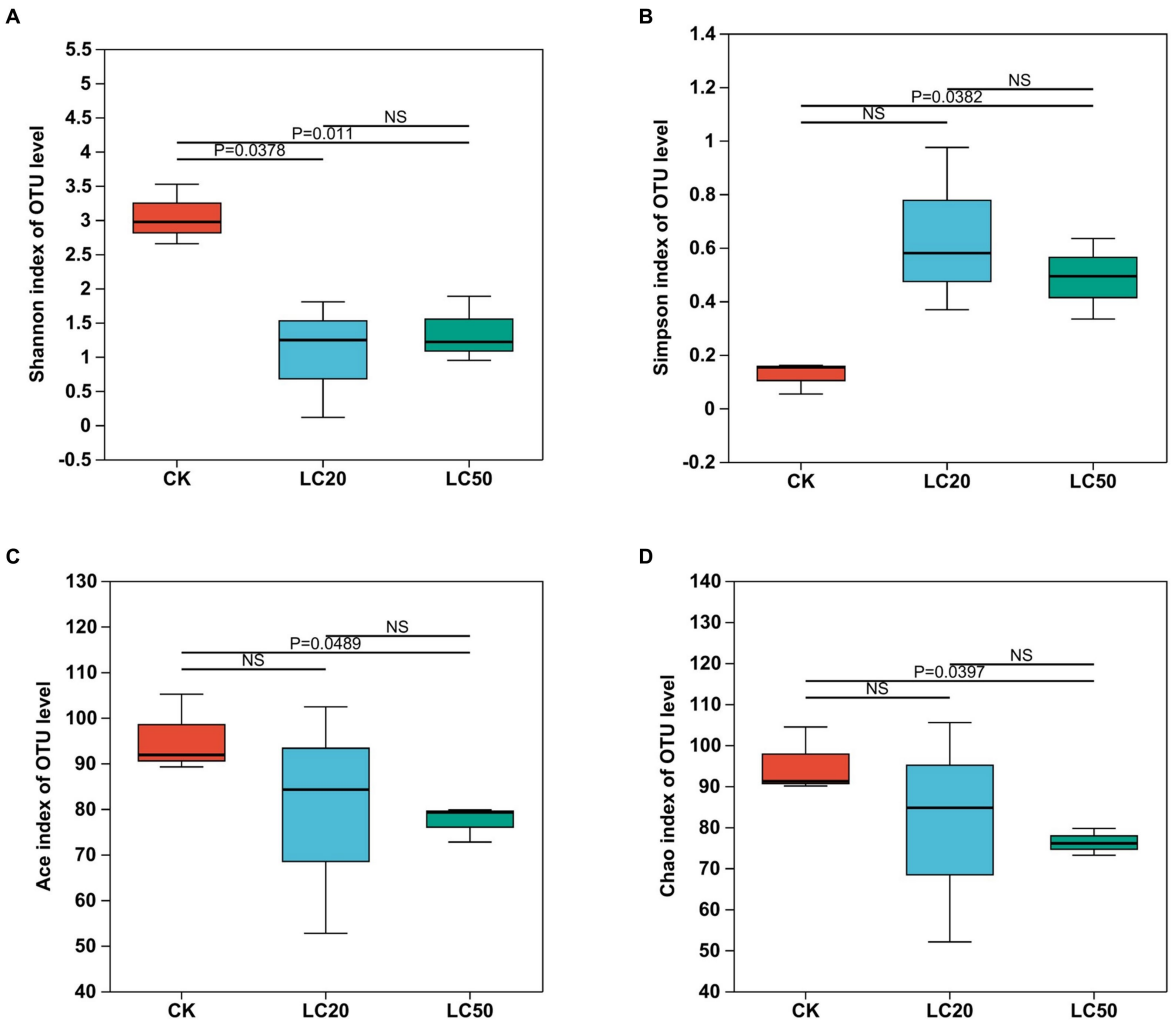
Effect of carvacrol treatment on the alpha diversity indices of gut bacterial in *L. dispar* larvae. Bacterial diversity is estimated by Shannon **(A)** and Simpson **(B)** indices. Bacterial richness is estimated by the Ace **(C)** and Chao1 **(D)** values. *p* ≤ 0.05 indicates significant difference; *p* ≤ 0.01 indicates extremely significant difference, NS indicates no significant difference. CK: control group; LC20: treatment group with 0.0297 mg/mL of CAR; LC50: treatment group with 1.120 mg/mL of CAR.

Seen from Figure 4, the Alpha diversity analysis of the bacterial composition in the midgut of the *L. dispar* revealed that the Shannon index in the two doses of CAR treatment groups was significantly lower than that in the control group (*p* < 0.05), indicating that the bacterial community diversity in the midgut of the *L. dispar* larvae treated with CAR was lower than that in the control group. In addition, the Simpson index in the LC50 treatment group was significantly higher than that in the control group (*p* < 0.05), indicating that CAR at LC50 dose altered concentration degree of dominant bacteria in the midgut of the larvae. At the same time, both ACE and Chao indices in the two carvacrol treatment groups were lower than those in the control group, but the differences were not significant.

**Figure 5:**
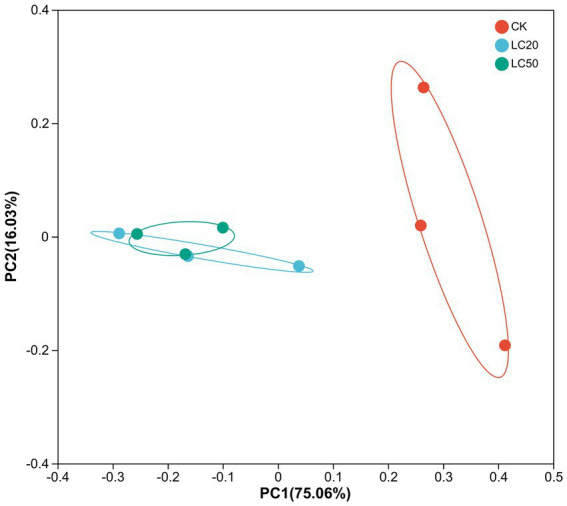
Principal coordinate analysis (PCoA) of bacterial communities in *L. dispar* from different groups based on unifrac distances. CK: control group; LC20: treatment group with 0.0297 mg/mL of CAR; LC50: treatment group with 1.120 mg/mL of CAR. The numbers 1–3 following the sample names represent different replicates, respectively.

In the PCoA analysis, the bacteria communities of the treatment group and the control group were significantly clustered in two different groups, and these variables together explained 91.09% of the total variance (Anosim, *R* = 0.449, *p* = 0.033; Adonis, *R^2^* = 0.652, *p* = 0.037) (Figure 5). The analysis of *β* diversity results showed that the gut bacterial composition of the CAR treatment group was different from that of the control group.

### 3.4 Functional prediction of the gut bacteria

**Figure 6:**
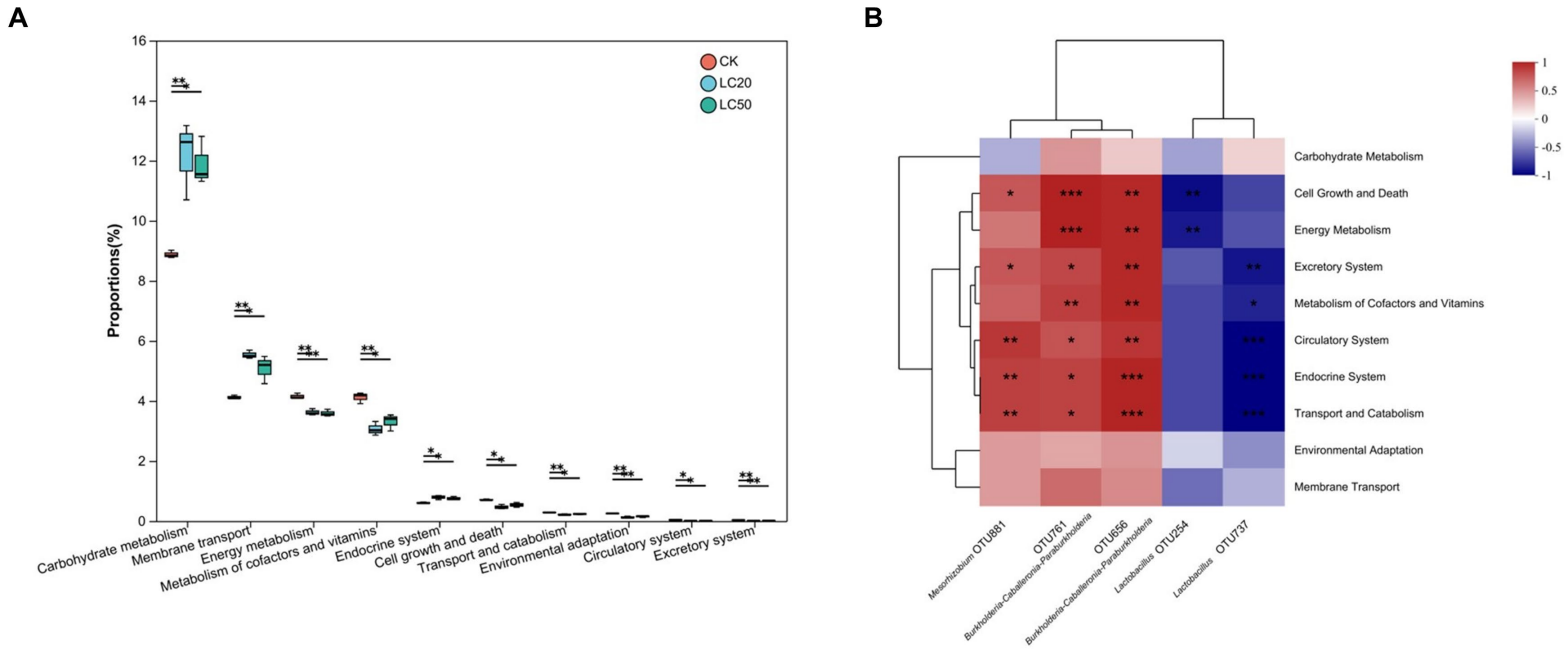
KEGG function prediction and function difference of the gut bacterial in *L. dispar* from different groups. **(A)** “*” indicates *p* < 0.05, “**” indicates *p* < 0.01. **(B)** “*” indicates that the correlation coefficient is greater than 0.7; “**” indicates that the correlation coefficient is greater than 0.8; “***” indicates that the correlation coefficient is greater than 0.9.CK: control group; LC20: treatment group with 0.0297 mg/mL of CAR; LC50: treatment group with 1.120 mg/mL of CAR. The numbers 1–3 following the sample names represent different replicates, respectively.

The 16S rRNA sequencing data were analyzed by PICRUSt to predict the function and pathway of gut bacteria in *L. dispar* larvae treated with two doses (LC20 and LC50) of carvacrol and the control group (Figure 6). Compared to the control group, the Carbohydrate metabolism and Membrane transport function of gut bacteria in the group treated with CAR significantly increased in the *L. dispar* larvae (*p* < 0.05). In addition, both doses of carvacrol treatment were able to significantly downregulate the functions of Energy metabolism, Signal transduction, Xenobiotics biodegradation and metabolism, Cell motility, Environmental adaptation, Circulatory system, Excretory system, Development and regeneration (*p* < 0.05). The LC50 dose treatment group was able to significantly downregulate the Metabolism of terpenoids and polyketides function of gut bacteria in *L. dispar* larvae (*p* < 0.05), but the effect of LC20 dose on could not significant downregulate the Metabolism of terpenoids and polyketides function (*p >* 0.05). Carvacrol had no significant effect on the Lipid metabolism function of gut bacteria in *L. dispar* larvae (*p* > 0.05).

To further investigate the relationship between OTUs and functions, we analyzed the correlation between OTU abundance and differential functional abundance. *Lactobacillus* (OTU 254 and OTU 737) was negative correlated with Cell Growth and Death, Energy Metabolism, Excretory System, Circulatory System, Endocrine System, Transport and Catabolism and Membrane Transport. The negative correlation coefficient between energy metabolism and OTU 254 was 0.81. *Mesorhizobium* (OTU881) and *Burkholderia-Caballeronia-Paraburkholderia* (OTU761 and OTU656) were positively correlated with Membrane Transport, Environmental Adaptation, Transport and Catabolism, Endocrine System, Circulatory System, Excretory System, Energy Metabolism, and Cell Growth and Death. The positive correlation coefficient between energy metabolism and OTU761 was 0.92. Overall, the principal functions of the majority of gut bacteria were associated with energy metabolism as well as cell growth and death.

### 3.5 Species relationships among gut bacteria

**Figure 7:**
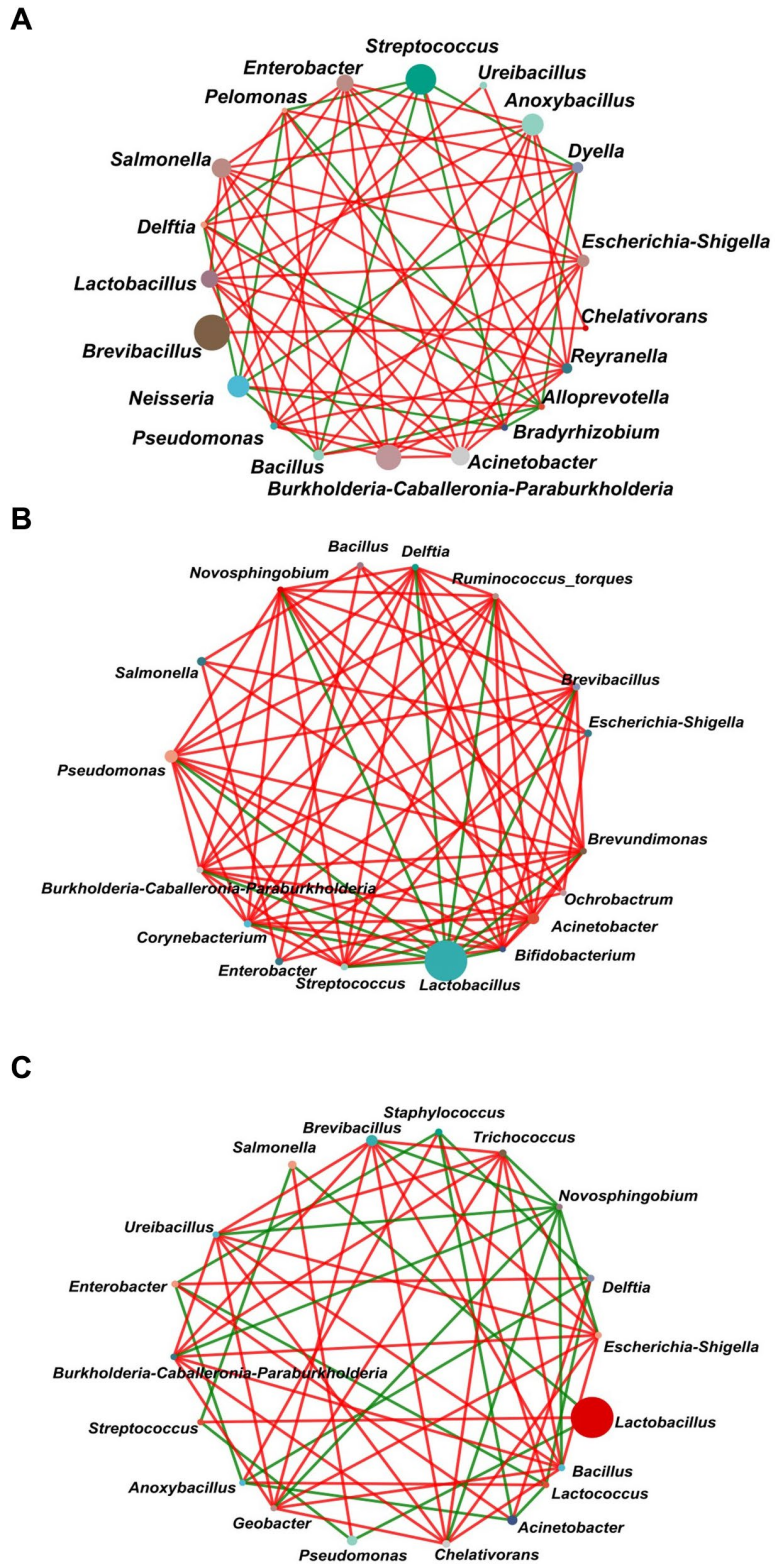
Network analysis of the dominant genera (top 20) of the gut bacterial communities of *L. dispar* in three groups. **(A)** CK: control group; **(B)** LC20: treatment group with 0.0297 mg/mL of CAR; **(C)** LC50: treatment group with 1.120 mg/mL of CAR. The size of nodes in the figure represents the abundance of species, and different colors represent different species. The color of the line indicates positive and negative correlation, red indicates positive correlation, green indicates negative correlation; The more lines there are, the more closely related the species is to other species. When a correlation coefficient exceeds 0.6 and *p* < 0.05, the relationships are kept.

In order to explore the interactions between the gut bacterial communities of *L. dispar* larvae treatment with carvacrol (the dose were 0.0297 mg/mL and 1.120 mg/mL separately), the control group was fed an artificial diet containing 10%DMSO, a single factor correlation network was used to analyze for the dominant genera in the top 20 genus of total abundance in each of the three groups (Figure 7). The network diagram for the control group included 20 nodes and 67 edges (52 positive correlations, and 15 negative correlations) (Figure 7A). There were 17 nodes and 76 edges (65 positive correlations, and 11 negative correlations) for the treatment group with 0.0297 mg/mL of CAR (Figure 7B). The network diagram for the LC_50_ group included 19 nodes and 57 edges (36 positive correlations, and 21 negative correlations) (Figure 7C). The number of nodes was almost the same in the three groups, and the LC_50_ treatment group had the least number of edges. In the control group the degree of *Lactobacillus* was eight, and both were positively correlated. However, in the LC_50_ carvacrol treated group, the degree of *Lactobacillus* was 3, respectively which were negatively correlated with *Streptococcus*. The results showed that under carvacrol stress of LC50, cooperation and exchange events between most bacterial genera in the gut of *L. dispar* were reduced.

### 3.6 The impact of gut bacteria elimination on the response of *Lymantria dispar* under CAR stress

**Figure 8:**
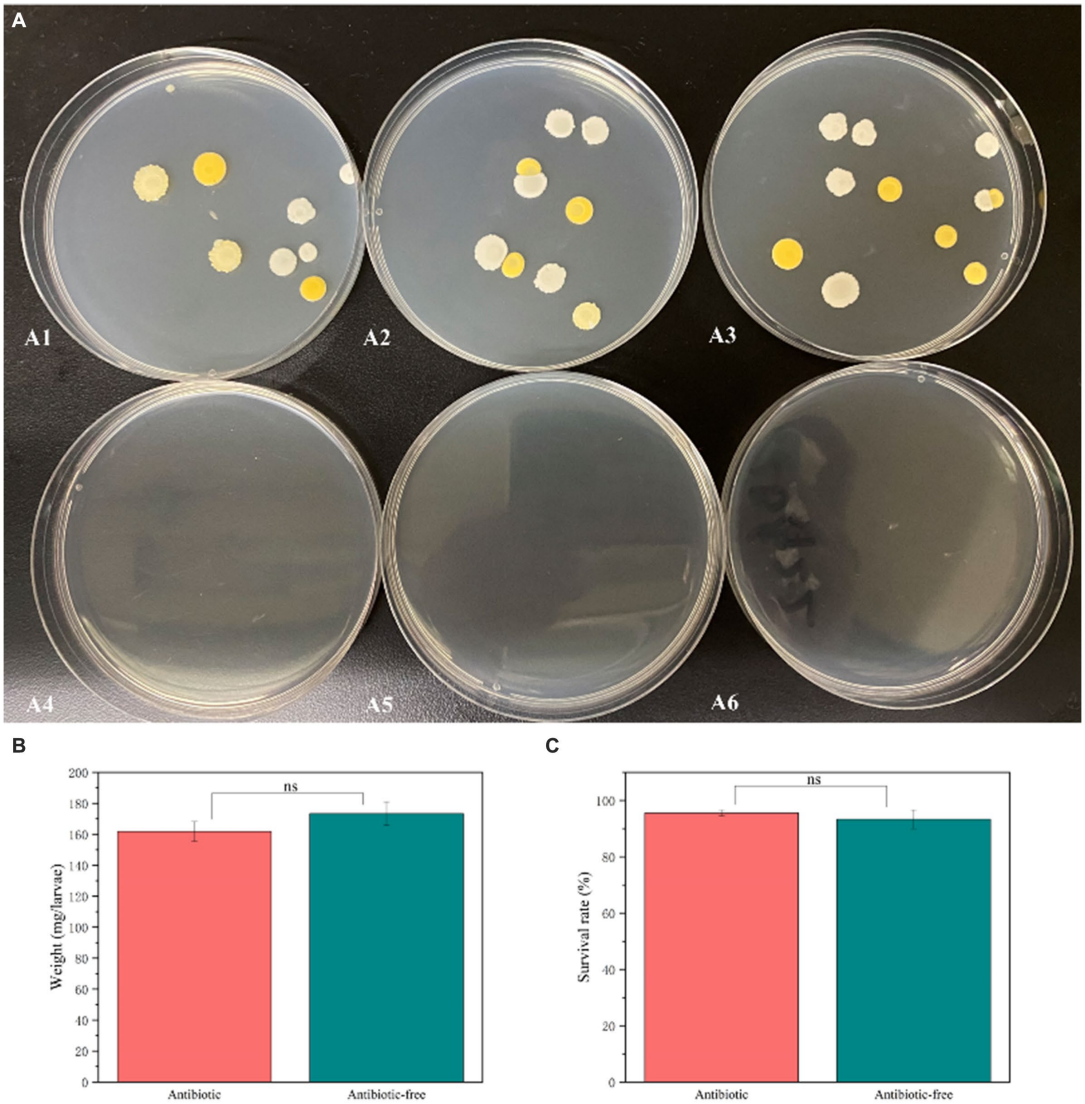
The impact of antibiotic treatment on the abundance of gut bacteria **(A)**, the increase in larval weight **(B)**, and survival rates **(C)** in *Lymantria dispar* larvae.

To further investigate the interaction between the gut bacteria of the *L. dispar* and CAR, we constructed an axenic insect system by incorporating antibiotics into the artificial diet, thereby validating the function of gut bacteria. Isolation and cultivation results of gut bacteria from *L. dispar* larvae after antibiotic treatment were shown in Figure 8A. After feeding on artificial feed containing antibiotics for 3 days, the abundance of gut bacteria in the larvae of the *L. dispar* (Figures 8A4–A6) was lower than that in the larvae which did not feed on antibiotic containing feed (Figures 8A1–A3). The results indicated that the method of incorporating antibiotics into artificial feed was effective in inhibiting the gut bacteria of the *L. dispar*. In addition, to determine whether antibiotics affect the growth and development of the *L. dispar* larvae, we measured the weight gain (Figure 8B) and survival rate (Figure 8C) of larvae fed on artificial feed containing antibiotics. Compared with the control group, antibiotic treatment had no significant effect on the growth and development of the larvae (*p* > 0.05). The results indicated that the *L. dispar* larvae could complete normal growth and development after gut bacteria were removed.

To further investigate the interaction between CAR and the gut bacteria of *L. dispar* larvae, we assessed the impact of CAR on the survival rate of the larvae which gut bacteria were removed. Compared with our previous research results, the insecticidal activity of carvacrol against *L. dispar* larvae was enhanced after feeding on artificial feed containing antibiotics at 72 h. The lethal concentration of carvacrol for *L. dispar* larvae with eliminated gut bacteria was 0.732 (0.604 ~ 0.902) mg/mL (Table 3), which was significantly higher than the lethal concentration for larvae with non-eliminated gut bacteria, which was 1.120 (0.918 ~ 1.415) mg/mL. The research results indicated that gut bacteria could significantly alter the response of *L. dispar* to carvacrol.

**Table 3:** Toxicity of carvacrol to *Lymantria dispar* larvae which gut bacteria were removed.

Treatment time (h)	LC_20_ (mg/mL)	CI (mg/mL)	LC_50_ (mg/mL)	CI (mg/mL)	*R* ^2^
72	0.183	(0.134 ~ 0.234)	0.732	(0.604 ~ 0.902)	0.971

## 4 Discussion

During the long-term coevolution between plants and herbivorous insects, various adaptive or defensive mechanisms have been formed, including morphological structure, physiological and biochemical metabolism. Plants can produce secondary metabolites including alkaloids and terpenes to resist the harm of herbivorous insects, while insects can resist the effects of exogenous toxins through their own physiological and biochemical defense mechanisms (Erb and Robert, 2016; Price, 2002). Additionally, a vast array of microorganisms was established within insects, distributed across the gut, bloodstream, and cells (Douglas, 2015). Notably, the density of gut microbiota is exceedingly high, and these microorganisms can significantly influence the detoxification process of their host insects (Douglas, 2015). Therefore, exploring the relationship between gut bacterial and their hosts was crucial for developing pest control strategies. Our preliminary studies indicated that carvacrol had toxicity on the *L. dispar* larvae and could affect the detoxification mechanisms at the mRNA level. However, there were few reports on the response of “an important organelle” which was gut bacteria under CAR stress. Consequently, we employed the 16 s rRNA technique to analyze the impact of sublethal and lethal doses of CAR on the composition, diversity, and function of the gut bacteria in the *L. dispar* larvae, with the aim of providing novel insights for the prevention and control of *L. dispar* larvae.

In this study, we found that under the treatment of CAR, the relative abundance, richness, diversity and function of gut bacterial in the larvae of the *L. dispar* had been significantly changed. The dominant bacteria at the phylum level of the *L. dispar* larvae were the *Proteobacteria* and the *Firmicutes*, accounting for over 97% of the gut bacteria of this species. This community was consistent with the findings of previous studies on the midgut bacteria of the *L. dispar* larvae (Zeng et al., 2021). At the genus level, we found that CAR treatment could significantly increase the abundance of *Lactobacillus* in the gut bacteria of *L. dispar* larvae (*p* = 0.01). As an important probiotic, *Lactobacillus* could produce a variety of probiotic effects on the host, including improving immune function, providing nutrients and pathogen exclusion (Blum et al., 2013; Ryu et al., 2008; Storelli et al., 2011). Studies had shown that *Lactobacillus* had a metabolic effect on Chlorpyrifos and could reduce the toxicity of Chlorpyrifos to *Drosophila melanogaster* (Daisley et al., 2018). *Lactobacillus* could significantly improve the survival rate of honeybees under acetamiprid stress (Liu et al., 2022). In addition, *Lactobacillus* could also help their hosts adapt to extreme low temperature environments (Liu et al., 2021).

According to the test of significant difference between groups, after CAR treatment, Significant declines occurred in the abundance of *Burkholderia-Caballeronia-Paraburkholderia*, *Pelomonas*, *Anoxybacillus*, and *Mesorhizobium* in the gut of the larvae. As a new taxonomic branch, the genus *Burkholderia-Caballeronia-Paraburkholderia* encompasses numerous beneficial bacterial species, which had been associated with diazotrophism, bioremediation, and antibiotic activity (Beukes et al., 2017). Upon infection with the cypovirus, the abundance of *Pelomonas* in the gut of the *Bombyx mori* had been significantly decreased (Sun et al., 2016). Similarly, in this study, it was observed that under the stress of CAR, the abundance of *Pelomonas* in the gut of the larvae also significantly declined. *Anoxybacillus* was a facultatively anaerobic, spore-forming Gram-positive bacterium, which was a new genus that had been independently separated from the *Bacillus* (Pikuta et al., 2000). Although only a few studies had reported the presence of *Anoxybacillus* within the gut of insects, it had been observed that *Anoxybacillus* has already been found in the gut of the glassy-winged sharpshooter larvae. Furthermore, *Mesorhizobium*, which belongs to the Phyllobacteriaceae family, was also present in the gut of *Triatoma infestans* (Waltmann et al., 2019).

As a plant secondary metabolite, carvacrol could inhibit the growth of microorganisms such as *Bacillus subtilis*, *Escherichia coli*, and *Pseudomonas aeruginosa* by interfering with energy metabolism and disrupting cell membranes (Chan et al., 2013; Wan et al., 2019). We also found that carvacrol could interfere with the energy metabolism and membrane transport functions of gut bacteria in the *L. dispar* larvae. Research had shown that Imidacloprid and Flupyradifurone could down-regulate the carbohydrate metabolism and up-regulate the energy metabolism function of the gut bacterial in *Bombus terrestris* (Zhang et al., 2022). Differently, we had observed a significant upregulation in carbohydrate metabolism a in the gut bacterial with two dose of CAR treatment (*p* < 0.05). The decrease in energy metabolism function of intestinal bacteria might be due to the bacteria consuming a large amount of energy to reduce the damage caused by CAR. The upregulation of carbohydrate metabolism function might be due to the increase in the relative abundance of *Lactobacillus* (Zhang et al., 2022).

The network analysis between microorganisms could clearly display the interactions between microorganisms (Wang et al., 2023). In the control group, the relationship between *Lactobacillus* and other bacterial genera was positively correlated. However, in the CAR treatment groups, the relationship between *Lactobacillus* and other bacterial genera was negatively correlated. *Lactobacillus*, as an important type of probiotic, exhibited multiple functions, including inhibiting the growth of pathogenic bacteria in the gut, improving the structure of gut bacteria, and promoting intestinal digestion (Lu et al., 2023). In this study, the alteration in the previously associated relationship between *Lactobacillus* and other genera may be attributed to the significant increase in the relative abundance of *Lactobacillus* in the gut. The insecticidal activity of carvacrol to *L. dispar* larvae eliminated by intestinal bacteria was higher than that *L. dispar* of larvae not eliminated by intestinal bacteria. Combined with the significant increase in *Lactobacillus* abundance under carvacrol stress. We speculated that *Lactobacillus* could help the larvae of the *L. dispar* adapt to the stress of carvacrol. The degradation capability of *Lactobacillus* toward carvacrol still was require further investigation.

## 5 Conclusion

The present study provided new insight into the effect of sublethal and lethal doses of carvacrol on the gut bacteria of *Lymantria dispar* larvae through 16S rRNA high-throughput sequencing technology. Findings indicated that carvacrol at both doses significantly affected the composition of gut bacterial at both the genus and phylum levels. Carvacrol could significantly affect the diversity, and function of gut bacteria. Carvacrol at both doses altered the interactions among gut bacteria. After carvacrol treatment, the survival rate of *L. dispar* larvae with gut bacteria eliminated was significantly higher than that of larvae without eliminated gut bacteria, and this effect was not caused by the antibiotics used to eliminate gut bacteria. Combined with the significant increase in the abundance of *Lactobacillus*, we speculated that gut bacteria mainly promoted the decomposition and metabolism of carvacrol in the larvae of *L. dispar* by increasing the abundance of *Lactobacillus*. The mechanism by which Lactobacillus alleviate the toxic effects of carvacrol on *L. dispar* will be explored in the next step of work. This study could provide insights into the role of gut bacteria in host resistance to harmful substance stress.

## Data Availability

The datasets presented in this study can be found in online repositories. The names of the repository/repositories and accession number(s) can be found at: https://www.ncbi.nlm.nih.gov/sra/PRJNA1155136.

## Footnotes

^1^https://github.com/OpenGene/fastp, version 0.20.0.

^2^http://www.cbcb.umd.edu/software/flash, version 1.2.7.

^3^http://drive5.com/uparse/, version 7.1.

^4^http://rdp.cme.msu.edu/, version 2.2.

^5^http://cloud.majorbio.com/

## References

[ref1] Bai J. Y. Xu Z. Li L. Ma W. Xu L. T. Ma L. (2020). Temporospatial modulation of *Lymantria dispar* immune system against an entomopathogenic fungal infection. Pest Manag. Sci. 76, 3982–3989. doi: 10.1002/ps.5947, PMID: 32506667

[ref2] Bai J. Y. Xu Z. Li L. Zhang Y. Diao J. Cao J. Y.. (2023). Gut bacterial microbiota of *Lymantria dispar* asiatica and its involvement in *Beauveria bassiana* infection. J. Invertebr. Pathol. 197:107897. doi: 10.1016/j.jip.2023.107897 36806463

[ref3] Beukes C. W. Palmer M. Manyaka P. Chan W. Y. Avontuur J. R. van Zyl E.. (2017). Genome data provides high support for generic boundaries in *Burkholderia Sensu Lato*. Front. Microbiol. 8:1154. doi: 10.3389/fmicb.2017.01154, PMID: 28694797 PMC5483467

[ref4] Blum J. E. Fischer C. N. Miles J. Handelsman J. (2013). Frequent replenishment sustains the beneficial microbiome of *Drosophila melanogaster*. MBio 4, e00860–e00813. doi: 10.1128/mBio.00860-13, PMID: 24194543 PMC3892787

[ref5] Briones-Robiero C. I. Hernández-García J. A. Gonzalez-Escobedo R. Soto-Robles L. V. Rivera-Orduña F. N. Zúñiga G. (2017). Structure and dynamics of the gut bacterial microbiota of the bark beetle, *Dendroctonus rhizophagus* (Curculionidae: Scolytinae) across their life stages. PLoS One 12:e0175470. doi: 10.1371/journal.pone.0175470 28406998 PMC5391025

[ref6] Broadbent A. B. Pree D. J. (1997). Resistance to insecticides in populations of *Frankuniella Occidentalis* (Pergande) (Thysanoptera: Thripidae) from greenhouses in the Niagara region of Ontario. Can. Entomol. 129, 907–913. doi: 10.4039/Ent129907-5

[ref7] Cao C. W. Sun L. L. Wen R. R. Shang Q. L. Ma L. Wang Z. Y. (2015). Characterization of the transcriptome of the Asian gypsy moth *Lymantria dispar* identifies numerous transcripts associated with insecticide resistance. Pestic. Biochem. Physiol. 119, 54–61. doi: 10.1016/j.pestbp.2015.02.005, PMID: 25868817

[ref8] Chan A. C. Ager D. Thompson I. P. (2013). Resolving the mechanism of bacterial inhibition by plant secondary metabolites employing a combination of whole-cell biosensors. J. Microbiol. Methods 93, 209–217. doi: 10.1016/j.mimet.2013.03.021 23566822

[ref9] Chang H. Guo J. L. Qi G. J. Gao Y. Wang S. W. Wang X. A.. (2023). Comparative analyses of the effects of sublethal doses of emamectin benzoate and tetrachlorantraniliprole on the gut microbiota of *Spodoptera frugiperda* (Lepidoptera: Noctuidae). J. Insect Sci. 23:7. doi: 10.1093/jisesa/iead039, PMID: 37471131 PMC10358434

[ref10] Chen Y. Z. Li T. Yang J. Li Q. M. Zhang G. C. Zhang J. (2022). Transcriptomic analysis of interactions between *Lymantria dispar* larvae and carvacrol. Pestic. Biochem. Physiol. 181:105012. doi: 10.1016/j.pestbp.2021.105012, PMID: 35082035

[ref11] Chen Y. Z. Zhang B. W. Yang J. Zou C. S. Li T. Zhang G. C.. (2021). Detoxification, antioxidant, and digestive enzyme activities and gene expression analysis of *Lymantria dispar* larvae under carvacrol. J. Asia Pac. Entomol. 24, 208–216. doi: 10.1016/j.aspen.2020.12.014

[ref12] Chen S. F. Zhou Y. Q. Chen Y. R. Gu J. (2018). Fastp: an ultra-fast all-in-one FASTQ preprocessor. Bioinformatics 34, i884–i890. doi: 10.1093/bioinformatics/bty560 30423086 PMC6129281

[ref13] Daisley B. A. Trinder M. McDowell T. W. Collins S. L. Sumarah M. W. Reid G. (2018). Microbiota-mediated modulation of organophosphate insecticide toxicity by species-dependent interactions with *lactobacilli* in a *Drosophila melanogaster* insect model. Appl. Environ. Microbiol. 84:e02820-17. doi: 10.1128/AEM.02820-17, PMID: 29475860 PMC5930343

[ref14] Douglas A. E. (2015). “Multiorganismal insects: diversity and function of resident microorganisms” in Annual review of entomology. ed. Berenbaum M. R., Vol, vol. 60, 17–34. 10.1146/annurev-ento-010814-020822 PMC4465791 25341109

[ref15] Edgar R. C. (2013). UPARSE: highly accurate OTU sequences from microbial amplicon reads. Nat. Methods 10, 996–998. doi: 10.1038/nmeth.2604, PMID: 23955772

[ref16] Erb M. Robert C. A. M. (2016). Sequestration of plant secondary metabolites by insect herbivores: molecular mechanisms and ecological consequences. Curr. Opin. Insect Sci. 14, 8–11. doi: 10.1016/j.cois.2015.11.005, PMID: 27436640

[ref17] Erler F. (2005). Fumigant activity of six monoterpenoids from aromatic plants in Turkey against the two stored-product pests confused flour beetle, *Tribolium confusum*, and Mediterranean flour moth, *Ephestia kuehniella*. J. Plant Dis. Protect. 112, 602–611. doi: 10.1007/BF03356158

[ref18] Fuentes C. Fuentes A. Barat J. M. Ruiz M. J. (2021). Relevant essential oil components: a minireview on increasing applications and potential toxicity. Toxicol. Mech. Methods 31, 559–565. doi: 10.1080/15376516.2021.1940408, PMID: 34112059

[ref19] Genta F. A. Dillon R. J. Terra W. R. Ferreira C. (2006). Potential role for gut microbiota in cell wall digestion and glucoside detoxification in *Tenebrio molitor* larvae. J. Insect Physiol. 52, 593–601. doi: 10.1016/j.jinsphys.2006.02.007, PMID: 16600286

[ref20] Jiang Y. J. Li S. Z. Li R. P. Zhang J. Liu Y. H. Lv L. F.. (2017). Plant cultivars imprint the rhizosphere bacterial community composition and association networks. Soil Biol. Biochem. 109, 145–155. doi: 10.1016/j.soilbio.2017.02.010

[ref21] Konecka E. Kaznowski A. Grzesiek W. Nowicki P. Czarniewska E. Baranek J. (2020). Synergistic interaction between carvacrol and *Bacillus thuringiensis* crystalline proteins against *Cydia pomonella* and *Spodoptera exigua*. BioControl 65, 447–460. doi: 10.1007/s10526-020-10011-4

[ref22] Konig I. Iftikhar N. Henry E. English C. Ivantsova E. Souders C. L. 2nd. (2023). Toxicity assessment of carvacrol and its acetylated derivative in early staged zebrafish (*Danio rerio*): safer alternatives to fipronil-based pesticides? Comp. Biochem. Physiol. C Toxicol. Pharmacol. 274:109762. doi: 10.1016/j.cbpc.2023.109762 37813296

[ref23] Langille M. G. I. Zaneveld J. Caporaso J. G. McDonald D. Knights D. Reyes J. A.. (2013). Predictive functional profiling of microbial communities using 16S rRNA marker gene sequences. Nat. Biotechnol. 31, 814–821. doi: 10.1038/nbt.2676, PMID: 23975157 PMC3819121

[ref24] Liu P. Niu J. Zhu Y. Li Z. Ye L. Cao H.. (2022). Apilactobacillus kunkeei alleviated toxicity of Acetamiprid in honeybee. Insects 13:1167. doi: 10.3390/insects13121167, PMID: 36555077 PMC9784809

[ref25] Liu P. Zhu Y. Ye L. Shi T. Li L. Cao H.. (2021). Overwintering honeybees maintained dynamic and stable intestinal bacteria. Sci. Rep. 11:22233. doi: 10.1038/s41598-021-01204-7, PMID: 34782655 PMC8593070

[ref26] Llana-Ruiz-Cabello M. Gutiérrez-Praena D. Pichardo S. Moreno F. J. Bermúdez J. M. Aucejo S.. (2014). Cytotoxicity and morphological effects induced by carvacrol and thymol on the human cell line Caco-2. Food Chem. Toxicol. 64, 281–290. doi: 10.1016/j.fct.2013.12.005, PMID: 24326232

[ref27] Lu J. T. Su X. Y. Yang Z. D. Hu P. (2023). The correlation between the gut microbiota of *Endoclita signifer* (Lepidoptera, Hepialidae) larvae and their host preferences. Insects 14:919. doi: 10.3390/insects14120919, PMID: 38132593 PMC10744147

[ref28] Ma Q. Li L. Y. Le J. Y. Lu D. L. Qiao F. Zhang M. L.. (2018). Dietary microencapsulated oil improves immune function and intestinal health in *Nile tilapia* fed with high-fat diet. Aquaculture 496, 19–29. doi: 10.1016/j.aquaculture.2018.06.080

[ref29] Magierowicz K. Górska-Drabik E. Sempruch C. (2019). The insecticidal activity of *Satureja hortensis* essential oil and its active ingredient-carvacrol against *Acrobasis advenella* (Zinck.) (Lepidoptera, Pyralidae). Pestic. Biochem. Physiol. 153, 122–128. doi: 10.1016/j.pestbp.2018.11.010, PMID: 30744885

[ref30] Magoc T. Salzberg S. L. (2011). FLASH: fast length adjustment of short reads to improve genome assemblies. Bioinformatics 27, 2957–2963. doi: 10.1093/bioinformatics/btr507, PMID: 21903629 PMC3198573

[ref31] Moazeni N. Khajeali J. Izadi H. Mahdian K. (2014). Chemical composition and bioactivity of *Thymus daenensis* Celak (Lamiaceae) essential oil against two lepidopteran stored-product insects. J. Essent. Oil Res. 26, 118–124. doi: 10.1080/10412905.2013.860412

[ref32] Pikuta E. Lysenko A. Chuvilskaya N. Mendrock U. Hippe H. Suzina N.. (2000). *Anoxybacillus pushchinensis* gen. Nov., sp. nov., a novel anaerobic, alkaliphilic, moderately thermophilic bacterium from manure, and description of *Anoxybacillus flavitherms* comb. nov. Int. J. Syst. Evol. Microbiol. 50, 2109–2117. doi: 10.1099/00207713-50-6-2109 11155986

[ref33] Price P. W. (2002). Resource-driven terrestrial interaction webs. Ecol. Res. 17, 241–247. doi: 10.1046/j.1440-1703.2002.00483.x

[ref34] Qin J. J. Li R. Q. Raes J. Arumugam M. Burgdorf K. S. Manichanh C.. (2010). A human gut microbial gene catalogue established by metagenomic sequencing. Nature 464, 59–65. doi: 10.1038/nature08821 20203603 PMC3779803

[ref35] Ryu J. H. Kim S. H. Lee H. Y. Bai J. Y. Nam Y. D. Bae J. W.. (2008). Innate immune homeostasis by the homeobox gene caudal and commensal-gut mutualism in *Drosophila*. Science 319, 777–782. doi: 10.1126/science.1149357, PMID: 18218863

[ref36] Siddiqui J. A. Khan M. M. Bamisile B. S. Hafeez M. Qasim M. Rasheed M. T.. (2022). Role of insect gut microbiota in pesticide degradation: a review. Front. Microbiol. 13:870462. doi: 10.3389/fmicb.2022.870462, PMID: 35591988 PMC9111541

[ref37] Song J. W. Jung J. M. Nam Y. Jung J. K. Jung S. Lee W. H. (2022). Spatial ensemble modeling for predicting the potential distribution of *Lymantria dispar asiatica* (Lepidoptera: Erebidae: Lymantriinae) in South Korea. Environ. Monit. Assess. 194:889. doi: 10.1007/s10661-022-10609-4, PMID: 36241949

[ref38] Srivastava V. Griess V. C. Keena M. A. (2020). Assessing the potential distribution of Asian gypsy moth in Canada: a comparison of two methodological approaches. Sci. Rep. 10:22. doi: 10.1038/s41598-019-57020-7, PMID: 31913334 PMC6949248

[ref39] Stackebrandt E. Goebel B. M. (1994). Taxonomic note: a place for DNA-DNA reassociation and 16S rRNA sequence analysis in the present species definition in bacteriology. Int. J. Syst. Evol. Microbiol. 44, 846–849. doi: 10.1099/00207713-44-4-846

[ref40] Storelli G. Defaye A. Erkosar B. Hols P. Royet J. Leulier F. (2011). *Lactobacillus plantarum* promotes *Drosophila* systemic growth by modulating hormonal signals through TOR-dependent nutrient sensing. Cell Metab. 14, 403–414. doi: 10.1016/j.cmet.2011.07.012, PMID: 21907145

[ref41] Sun Z. L. Lu Y. H. Zhang H. Kumar D. Liu B. Gong Y. C.. (2016). Effects of BmCPV infection on silkworm *Bombyx mori* intestinal bacteria. PLoS One 11:e0146313. doi: 10.1371/journal.pone.0146313 26745627 PMC4706323

[ref42] Sun L. L. Wang Z. Y. Zou C. S. Cao C. W. (2014). Transcription profiling of 12 Asian gypsy moth (*Lymantria dispar*) cytochrome P450 genes in response to insecticides. Arch. Insect Biochem. Physiol. 85, 181–194. doi: 10.1002/arch.21152, PMID: 24488622

[ref43] Tong F. Gross A. D. Dolan M. C. Coats J. R. (2013). The phenolic monoterpenoid carvacrol inhibits the binding of nicotine to the housefly nicotinic acetylcholine receptor. Pest Manag. Sci. 69, 775–780. doi: 10.1002/ps.3443, PMID: 23255497

[ref44] Waltmann A. Willcox A. C. Balasubramanian S. Mayori K. B. Guerrero S. M. Sanchez R. S. S.. (2019). Hindgut microbiota in laboratory-reared and wild *Triatoma infestans*. PLoS Negl. Trop. Dis. 13:e0007383. doi: 10.1371/journal.pntd.0007383 31059501 PMC6522061

[ref45] Wan C. P. Shen Y. T. Nisar M. F. Qi W. W. Chen C. Y. Chen J. Y. (2019). The antifungal potential of carvacrol against *Penicillium Digitatum* through ^1^H-NMR based metabolomics approach. Appl. Sci. (Basel) 9:2240. doi: 10.3390/app9112240

[ref46] Wang Q. Garrity G. M. Tiedje J. M. Cole J. R. (2007). Naive Bayesian classifier for rapid assignment of rRNA sequences into the new bacterial taxonomy. Appl. Environ. Microbiol. 73, 5261–5267. doi: 10.1128/AEM.00062-07, PMID: 17586664 PMC1950982

[ref47] Wang S. C. Wang L. Y. Fan X. Yu C. Feng L. Yi L. (2020). An insight into diversity and functionalities of gut microbiota in insects. Curr. Microbiol. 77, 1976–1986. doi: 10.1007/s00284-020-02084-2 32535651

[ref48] Wang X. F. Wang H. L. Zeng J. Y. Cui Z. Z. Geng S. L. Song X. F.. (2023). Distinct gut bacterial composition in *Anoplophora glabripennis* reared on two host plants. Front. Microbiol. 14:1199994. doi: 10.3389/fmicb.2023.1199994 37405158 PMC10315502

[ref49] Wu H. Zheng L. Tan M. Li Y. Xu J. Yan S.. (2022). Cd exposure-triggered susceptibility to *Bacillus thuringiensis* in *Lymantria dispar* involves in gut microbiota dysbiosis and hemolymph metabolic disorder. Ecotoxicol. Environ. Saf. 241:113763. doi: 10.1016/j.ecoenv.2022.113763, PMID: 35696962

[ref50] Xu Z. Bai J. Y. Zhang Y. Li L. Min M. R. Cao J. Y.. (2023). Chromosome-level genome assembly of the Asian spongy moths *Lymantria dispar asiatica*. Sci. Data 10:898. doi: 10.1038/s41597-023-02823-7, PMID: 38092795 PMC10719281

[ref51] Youssefi M. R. Tabari M. A. Esfandiari A. Kazemi S. Moghadamnia A. A. Sut S.. (2019). Efficacy of two Monoterpenoids, Carvacrol and thymol, and their combinations against eggs and larvae of the West Nile vector *Culex pipiens*. Molecules 24:1867. doi: 10.3390/molecules24101867, PMID: 31096594 PMC6572342

[ref52] Zeng J. Y. Guo J. X. Shi J. H. Shi Z. B. Zhang G. C. Zhang J. (2021). Stress response of *Lymantria dispar asiatica* (Lepidoptera: Erebidae) larvae and its gut microbiota to manganese ion. J. For. Res. 32, 1241–1251. doi: 10.1007/s11676-020-01160-4

[ref53] Zeng J. Y. Shi J. H. Guo J. X. Shi Z. B. Zhang G. C. Zhang J. (2020a). Variation in the pH of experimental diets affects the performance of *Lymantria dispar asiatica* larvae and its gut microbiota. Arch. Insect Biochem. Physiol. 103:e21654. doi: 10.1002/arch.21654, PMID: 31916310

[ref54] Zeng J. Y. Shi Z. B. Shi J. H. Guo J. X. Zhang G. C. Zhang J. (2019). Ambient temperature-mediated enzymic activities and intestinal microflora in *Lymantria dispar* larvae. Arch. Insect Biochem. Physiol. 102:e21597. doi: 10.1002/arch.21597, PMID: 31328829

[ref55] Zeng J. Y. Wu D. D. Shi Z. B. Yang J. Zhang G. C. Zhang J. (2020b). Influence of dietary aconitine and nicotine on the gut microbiota of two lepidopteran herbivores. Arch. Insect Biochem. Physiol. 104:e21676. doi: 10.1002/arch.21676, PMID: 32323892

[ref56] Zhang C. S. Liu P. Sun L. L. Cao C. W. (2023). Integration of miRNA and mRNA expression profiles in Asian spongy moth *Lymantria dispar* in response to cyantraniliprole. Pestic. Biochem. Physiol. 191:105364. doi: 10.1016/j.pestbp.2023.105364 36963953

[ref57] Zhang Q. C. Wang Q. L. Zhai Y. F. Zheng H. Wang X. F. (2022). Impacts of Imidacloprid and Flupyradifurone insecticides on the gut microbiota of *Bombus terrestris*. Agriculture (Basel) 12:389. doi: 10.3390/agriculture12030389

[ref58] Zhao G. Liu W. Brown J. M. Knowles C. O. (1995). Insecticide resistance in field and laboratory strains of Western flower Thrips (Thysanoptera: Thripidae). J. Econ. Entomol. 88, 1164–1170. doi: 10.1093/jee/88.5.1164

